# Hypoxia and the Kynurenine Pathway: Implications and Therapeutic Prospects in Alzheimer's Disease

**DOI:** 10.1155/2021/5522981

**Published:** 2021-11-10

**Authors:** Oluyomi Stephen Adeyemi, Oluwakemi Josephine Awakan, Lawrence Boluwatife Afolabi, Damilare Emmanuel Rotimi, Elizabeth Oluwayemi, Chiagoziem A. Otuechere, Omodele Ibraheem, Tobiloba Chritiana Elebiyo, Omokolade Alejolowo, Afolake T. Arowolo

**Affiliations:** ^1^SDG 03 Group – Good Health & Well-being, Landmark University, Omu-Aran 251101, Kwara State, Nigeria; ^2^Department of Biochemistry, Medicinal Biochemistry, Nanomedicine & Toxicology Laboratory, Landmark University, PMB 1001, Omu-Aran-251101, Nigeria; ^3^Department of Biochemistry, Redeemer's University, Ede, Nigeria; ^4^Department of Biochemistry, Federal University of Oye-Ekiti, Oye Ekiti, Nigeria; ^5^Hair and Skin Research Laboratory, Division of Dermatology, Department of Medicine, Faculty of Health Sciences and Groote Schuur Hospital and Division of Medical Biochemistry, Department of Integrative Biomedical Sciences, University of Cape Town, Cape Town, Anzio Road Observatory 7925, South Africa

## Abstract

Neurodegenerative diseases (NDs) like Alzheimer's disease, multiple sclerosis, amyotrophic lateral sclerosis, Parkinson's disease, and Huntington's disease predominantly pose a significant socioeconomic burden. Characterized by progressive neural dysfunction coupled with motor or intellectual impairment, the pathogenesis of ND may result from contributions of certain environmental and molecular factors. One such condition is hypoxia, characterized by reduced organ/tissue exposure to oxygen. Reduced oxygen supply often occurs during the pathogenesis of ND and the aging process. Despite the well-established relationship between these two conditions (i.e., hypoxia and ND), the underlying molecular events or mechanisms connecting hypoxia to ND remain ill-defined. However, the relatedness may stem from the protective or deleterious effects of the transcription factor, hypoxia-inducible factor 1-alpha (HIF-1*α*). The upregulation of HIF-1*α* occurs in the pathogenesis of most NDs. The dual function of HIF-1*α* in acting as a “killer factor” or a “protective factor” depends on the prevailing local cellular condition. The kynurenine pathway is a metabolic pathway involved in the oxidative breakdown of tryptophan. It is essential in neurotransmission and immune function and, like hypoxia, associated with ND. Thus, a good understanding of factors, including hypoxia (i.e., the biochemical implication of HIF-1*α*) and kynurenine pathway activation in NDs, focusing on Alzheimer's disease could prove beneficial to new therapeutic approaches for this disease, thus the aim of this review.

## 1. Introduction

Neurodegeneration is the gradual loss of neuronal function coupled with the partial or total loss of cerebral and body coordination. Diseases associated with this disorder include Alzheimer's disease (AD), Parkinson's disease (PD), Huntington's disease (HD), multiple sclerosis (MS), and amyotrophic lateral sclerosis (ALS) [1]. In most cases, the underlying mechanisms behind these neurodegenerative diseases (NDs) remain elusive. Several morphological and pathological studies suggest that NDs may arise due to mitochondrial dysfunction, genetic predisposition, and environmental factors [2, 3].

There is a consensus that hypoxia (characterized by diminished tissue or cellular oxygen) correlates positively with the development of NDs [4]. However, the direct relationship between hypoxia and ND is not well understood. Evidence suggests that the presence of the transcription factor subunit, hypoxia-inducible factor 1-alpha (HIF-1*α*), could be the link between hypoxia and ND [5]. HIF-1*α*, a master regulator of the cellular/tissue response to hypoxia, plays dual roles by acting as a “killer factor” and “protective transcription factor” depending on the severity of the disease(s) causing the hypoxic condition [6]. Besides hypoxia, hyperactivity of the kynurenine pathway correlates positively with ND pathogenesis [2, 7–9]. The kynurenine pathway (KP) is a metabolic pathway involved in converting tryptophan to kynurenine. Apart from kynurenine synthesis, the KP also functions in immune and neurotransmission functions [10]. The KP's rate-limiting step consists of the indoleamine 2,3-dioxygenase (IDO) enzymatic catalysis of tryptophan breakdown to N-formylkynurenine (NFK). The further conversion of NFK to neurotoxic metabolites (like 3-hydroxykynurenine and quinolinic acid (QA)) aids ND development.

To date, there is no definitive cure for NDs despite the enormous efforts made by researchers in this field. Thus, this review discusses the factors that influence NDs, with a specific focus on AD, and highlights the roles of HIF-1*α* and the kynurenine pathway as potential therapeutic targets toward the discovery of novel treatments.

## 2. Factors Influencing Neurodegeneration

**Figure 1:**
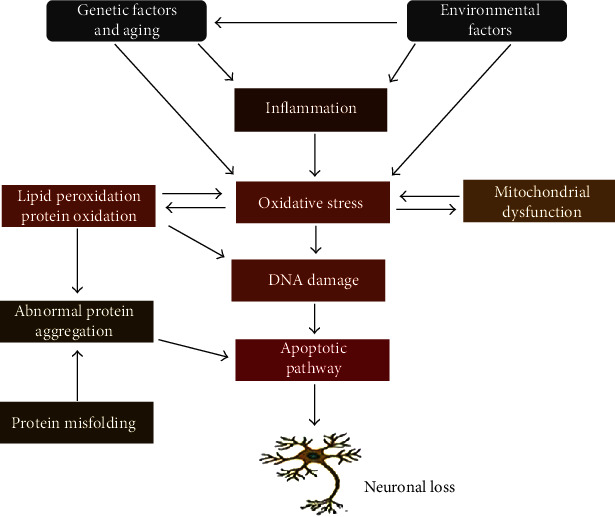
A schematic depiction of factors and cellular events involved in neurodegeneration. Factors such as aging, genetics, and environmental factors trigger neuronal loss via inflammation, oxidative stress, mitochondrial dysfunction, and abnormal protein aggregation.

Several factors that trigger neurodegeneration, such as genetic risk, aging, and environmental factors, may ultimately lead to neuronal death (Figure 1). Additionally, systemic inflammation can result in microglial activation linked to chronic neurodegeneration [11]. For instance, high proinflammatory immunoregulatory proteins are observed in the cerebrospinal fluid of most patients with ND [12]. Also, the imbalance in reactive oxygen species (ROS) produced results in oxidative stress and dysfunction in axoplasmic transport and, eventually, neuronal cell death [13]. Furthermore, compromised redox homeostasis resulting from hypoxia and kynurenine pathway activation [14, 15] plays a role in neurodegeneration [16, 17]. Discussed below are the major factors or conditions contributing to neurodegeneration.

### 2.1. Hereditary and Genetics

Nearly 70% of ND cases are related to genetic factors with the involvement of many specific genes, for example, in AD, amyloid precursor protein (APP), presenilin-1 (PSEN1), and presenilin-2 (PSEN2) genes. The mutations in any of these three genes may cause an early familial onset of AD. Also, mutations in more than 20 genes (PRKN, UCHL1, PARK7, LRRK2, PINK1, POLG, HTRA2, SYNJ1, DNAJC13, TMEM230, VPS13C, LRP10, ATP13A2, FBXO7, GIGYF2, GBA, PLA2G6, EIF4G1, VPS35, and DNAJC6) are associated with this disease, most of which are highly penetrant and often cause early-onset or atypical symptoms [18]. Gene mutation is also known to directly or indirectly affect oxidative stress via modulation of other influencing factors such as the impairment in mitochondrial function, protein misfolding, and microglial activation [19].

### 2.2. Mitochondrial Dysfunction

Mitochondrial dysfunction occurs in most neurodegenerative diseases. Several essential genes, including PARK7 (encoding DJ-1), *α*-synuclein, parkin, PINK1, or LRRK2, have pathogenic mutations in PD, which cause defects in mitochondrial dynamics and function. Meanwhile, PINK1 deletion results in increased oxidative stress within mitochondria [18]. AD defining the appearance of amyloid-*β* (A*β*) aggregates and tau pathologies correlates with mitochondrial dysfunction in neurons. Elevated Ca^2+^ and ROS levels during mitochondrial dysfunction contribute to the accumulation of tau protein aggregates [20].

### 2.3. Oxidative Stress

Although there is a strong association of oxidative stress resulting from highly reactive oxygen species (ROS) with neuronal death [21], it is difficult to establish if oxidative stress is solely responsible for neuronal death in neurodegenerative disorders. The cascade of unstable reactions involving ROS includes DNA oxidation, lipid peroxidation, and protein oxidation. Furthermore, these reactions lead to electron loss in the DNA and protein structures and ultimately damage the mitochondrial protein and DNA. This damage may create a pathway to neurodegeneration [22]. Oxidative stress, characterized by the overproduction of reactive oxygen species, induces mitochondrial DNA mutations, damages the mitochondrial respiratory chain, alters membrane permeability, and influences Ca^2+^ homeostasis and mitochondrial defense systems. ROS generated via exogenous and endogenous sources are superoxide ions, hydrogen peroxide, hydroxyl ions, and singlet oxygen [23]. Environmental toxicants such as pesticides are exogenous sources of ROS, while endogenous sources result from the endoplasmic reticulum and mitochondrial enzymes. A balanced cellular ROS level plays a vital role in regulating cellular signaling necessary for cell survival. Therefore, an imbalance in ROS homeostasis results in protein misfolding and DNA damage. Hence, imbalanced redox homeostasis amplifies neuronal dysfunction and triggers neurodegeneration, leading to the development of these neurodegenerative diseases [23].

### 2.4. Heavy Metals

Heavy metals (e.g., mercury and lead) may play a role in the pathogenesis of AD. These metals extend A*β* deposition and tau protein phosphorylation that characterizes AD [24]. Manganese and toxic solvents are also associated with PD features, including the accumulation of *α*-synuclein and impaired mitochondrial function, although metals are crucial in biological reactions as cofactors; however, dysregulation in homeostasis leads to ROS generation. For example, the increased cellular iron concentration may lead to an elevated oxidative stress state. The neurotoxin, 6-hydroxydopamine (6-OHDA), exemplifies this phenomenon. 6-Hydroxydopamine (6-OHDA) releases iron from ferritin, which leads to increased lipid peroxidation. Deferoxamine (DFO), an iron chelator, inhibits this reaction [25] and also upregulates HIF-1*α* [26]. HIF-1*α* is a crucial regulator of hypoxia; overexpression of HIF-1*α* is an essential factor to show aggressive phenotypes under hypoxic conditions [27].

### 2.5. Protein Misfolding and Aggregation

A hallmark of NDs is the accumulation of misfolded or aggregated proteins [28]. Protein functionality rests on the 2D amino acid sequence's proper folding to an energy-favorable 3-dimensional structural conformation of that protein [29]. In other words, protein misfolding arising from external factors, including aging and oxidative stress, results in the formation of protein aggregates and, ultimately, protein dysfunction [30].

### 2.6. Inflammation

Neuroinflammation triggers the onset of several neurodegenerative disorders. Several damage signals appear to induce neuroinflammation, such as trauma, infection, oxidative agents, redox iron, oligomers of tau, and A*β* [31]. In effect, neuroinflammation causes abnormal secretion of proinflammatory cytokines that trigger signaling pathways that activate tau hyperphosphorylation in residues not modified under normal physiological conditions [32].

## 3. Pathophysiology of Alzheimer's Disease

Alzheimer's disease (AD), named after a German Physician, is marked by the development of multiple cognitive deficits such as impaired memory, inability to initiate and plan complex behaviors, and aphasia [33]. AD is the most common form of dementia. The estimated global prevalence rate for AD will surpass 100 million patients by 2050 [34]. This situation will not only create a social burden but also increase the economic burden worldwide. In 2010, there was a worldwide estimated burden of 46.8 million people having dementia with care costs of about US$818 billion. By 2030, the number of people with dementia should exceed 74.7 million, with a caring cost of US$2 trillion [35]. Despite the vast number of scientific reports on AD, pharmacological prevention remains a challenge, although lifestyle changes (e.g., exercise, social and mental stimulation) could be effective preventive measures.

## 4. Key Players in the Pathophysiology of Alzheimer's Disease

### 4.1. Amyloid Precursor Protein

Amyloid precursor protein (APP) is an abundant type 1 integral transmembrane protein in the central nervous system. It is ubiquitously expressed in human tissues and localizes at the plasma membrane and organelles, such as the mitochondria, Golgi apparatus, and endoplasmic reticulum [36, 37]. Proteolytic processing of the synaptic protein APP produces a 40- or 42-amino acid protein fragment, A*β*, the chief component of amyloid plaques [38]. The most abundant amyloid-*β* protein among others is A*β*40 and A*β*42 in the brain; the only difference between A*β*40 and A*β*42 is the presence of isoleucine and alanine amino acid residues at the C-terminus of A*β*42 [39]. There is a preferential production of A*β* in the aforementioned cellular organelles based on the protein needs by both the amyloidogenic and nonamyloidogenic pathways (the secretory pathway) [40].

**Figure 2:**
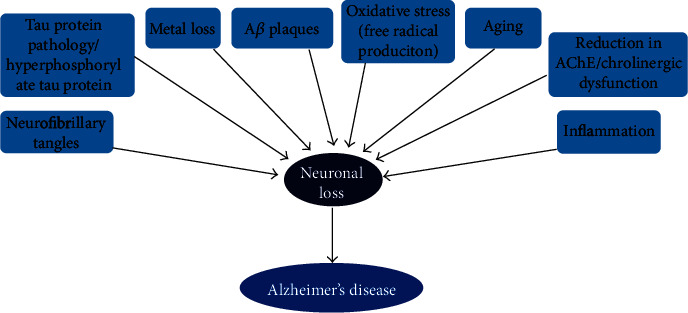
Schematic diagram showing some contributory factors to the pathology of AD. The mechanism by which amyloid-beta and neurofibrillary tangles are deposited in the brain to cause neuronal loss is unknown. Several hypotheses postulate neuronal loss in AD; however, amyloid-beta accumulation in the brain triggers a series of complex reactions that result in neuronal loss. Affected regions of the brain demonstrate inflammation, amyloid plaques, and neurofibrillary tangles.

The formation of A*β* oligomers results from the release of A*β* species in monomers, which then aggregates to form the amyloid plaques [41]. The A*β* oligomers trigger proinflammatory cascades, oxidative stress and mitochondrial impairment, induction of neuronal apoptosis, increased phosphorylation of tau proteins, deregulation of calcium metabolism, and cell death by interacting with neurons and glial cells (Figure 2) [42], making them the most toxic of all known amyloid derivatives. These have led to an impairment in APP metabolism due to a feedback loop that causes damage to neuronal cells.

### 4.2. Tau Protein

Tau protein belongs to a group of proteins referred to as microtubule-associated proteins (MAPs) and mainly expressed in neurons and plays a crucial role in neuronal cytoskeleton stabilization [43]. Phosphorylated tau proteins and isoforms interact with tubulins and stabilize microtubulin polymers. It also helps recruit signaling proteins and regulate microtubule-mediated axoplasmic transport or flow [44]. Furthermore, neuronal polarity depends on the microtubular properties of the dendrite and axon [45]. The mechanism of tau phosphorylation brings about synapse plasticity through cytoskeleton remodeling. Although the tau protein phosphorylation is essential, it requires tight control. Overphosphorylation will lead to neuronal death and disruption of the microtubular cytoskeleton. Therefore, the hyperphosphorylation of tau proteins causes axoplasmic transport dysfunction and impaired synaptic metabolism.

Tau phosphorylation and dephosphorylation thus serve as the regulatory point for neural homeostasis of serine-threonine phosphoepitopes by serine/threonine-protein kinase N1 (PKN). Here, phosphatase binds to guanine protein-coupled receptors (GPCRs) and ion channels, thus reversing protein phosphorylation [43]. Tau protein can be in insoluble and soluble forms. The insoluble form found in PHF (paired helical filaments) is the primary constituent of neurofibrillary tangles (NFTs), while the soluble form may devastate structural plasticity. There are six isoforms of tau in humans based on the number of tubulin-binding domains and differences in the N-terminus of the protein [45]. The dynamic polymerization of tau proteins occurs via tau and tubulin interaction, controlled by the phosphorylation and dephosphorylation of PKN. The longest tau protein isomer is said to have about 79 potential phosphorylation sites at threonine (Thr) and serine (Ser) residues, with an average of 30 in a normal isomer [36]. Phosphorylation and dephosphorylation increase conformational changes that affect tau interaction with *α*- and *β*-tubulins and stabilize microtubules [46]. The phosphorylation of tau proteins occurs with numerous proteases and protein kinases with an essential tau kinase in the neurons, glycogen synthase kinase-3*β* (GSK3*β*). The expression of protein phosphatases (PP1, PP2A, and PP5) is limited in the cerebral tissues of patients who have AD [47]. Hyperphosphorylation of tau protein by phosphoepitopes in PHF occurs in AD patients' brains. Some proline-directed kinases like casein kinases I and II, protein kinases A and C, calcium/calmodulin kinase II are present in NFTs and are central in regulating the action of neurofibers [47, 48]. During the embryonic developmental stage of the CNS, the neuronal tau protein is mainly in a hyperphosphorylated state because there is a high demand for neuroplastic changes in neurons and synapses [48].

Contrary to the developmental stage of the CNS, there is maintenance in the stability of neuronal homeostasis in the matured stage because tau phosphoepitopes are predominantly in a dephosphorylated state [49]. However, the neuronal process outgrowth and synaptic plasticity are maintained continuously by the changes in tau phosphorylation. There is an abnormality in tau hyperphosphorylation in some pathologic conditions like AD, causing impairment in its binding to tubulin, destabilizing the microtubular structure. This impairment also leads to synaptic metabolism and axoplasmic transport dysfunction, resulting in cytoskeleton collapse and neural death [50].

### 4.3. The Amyloid Cascade

The amyloid cascade hypothesis of AD was initially reported in the year 1992 [51]. This theory postulated that the enhanced aggregation of A*β* peptides into neuritic and senile plaques in the brain triggers neuronal degradation involving impaired mitochondrial function, decreased neurogenesis and synaptic plasticity, free radical generation, tau protein hyperphosphorylation, and impaired calcium metabolism in AD [52]. However, recent studies have shown that A*β* peptides act to trigger the amyloid cascade and promote fibrillary and oligomeric forms (the most toxic forms of A*β* peptides) [53].

The genetic mutations in early-onset familial AD strengthen the *in vivo* and *in vitro* findings on the amyloid cascade hypothesis. Consequently, harnessing the tool of gene engineering created AD animal models bearing these mutations [54]. The amyloid hypothesis of AD came with several cautions, such as the fact that no significant correlation exists between chronic dementia and the density of amyloid plaques in the brain. Neuritic plaques, also called senile plaques, are extracellular deposits of A*β* peptides enclosed by reactive astrocytes and activated microglia in the brain's gray matter associated with neurodegeneration [55]. In contrast, AD is considered the sole neurodegenerative disease in which the A*β* peptide is the pathological cause, as revealed by the statistics of nondemented elderly individuals who have amyloid plaques in their brain following necropsy examination. Also, there are cases of plaque counts in nondemented subjects corresponding to those detected in AD patients [56]. Secondly, clinical trials showed that anti-amyloid-based therapeutic drugs and strategies failed to combat dementia progression or improve cognitive processing [56]. Finally, AD's early onset has been AD based on the genetic mutations proven by AD cellular and animal models. However, early-onset AD records a smaller percentage of cases of dementia, while late-onset AD is far more frequent and has no relationship with genetic mutations. Contrary to that, sporadic AD has a multifactorial origin involving numerous genetic polymorphisms with fewer risk effects, pathogenic amyloid, and other pathological mechanisms [57].

### 4.4. The Cholinergic Hypothesis

Over the years, the cholinergic hypothesis has been the main postulation for neurodegenerative disorders. All cholinergic neurons use the neurotransmitter acetylcholine (AChE). AChE is an *α*/*β*-fold protein produced in the cell from acetyl-CoA and choline. Transported into the synapse through microtubules, AChE binds to a fast nicotinic receptor and a slow muscarinic receptor. AChE is involved in the consolidation/reconsolidation and retrieval of memory. This role of AChE lends credence to studies showing that acetylcholine diminishes in individuals with neurodegenerative disorders such as AD [58, 59]. The cholinergic hypothesis proposed that acetylcholine changes the conformation of NFTs in the brain of AD patients through noncholinergic function by amyloid-beta deposition [60]. Furthermore, the literature suggests that the cholinergic neurons' degeneration from the nucleus basalis of Meynert plays a crucial role in the memory loss experienced by AD patients [59].

## 5. The Role of Hypoxia in the Progression of Alzheimer's Disease

A limited supply of oxygen to the tissues results in hypoxia. The heart pumps oxygenated blood to the periphery and is crucial for cellular/tissue/organ function and oxidative phosphorylation performance. Hypoxia occurs by several mechanisms, including respiratory system failure, inadequate hemoglobin production, chemical induction of hypoxia, or inadequate blood flow to an organ [61]. The stabilization of the HIFs (hypoxia-inducible factors) controls the hypoxia signaling pathway, which is activated by hypoxia. HIF protein is degraded by von Hippel-Lindau protein (pVHL), an E3 ubiquitin ligase, when it binds to the hydroxylated HIF-*α* acting as a substrate recognition element of the E3 ubiquitin ligase complex. On the other hand, factors inhibiting HIF-*α* (FIHs) hydroxylate the asparagine residues of HIF-*α* subunits, which then inhibits the binding of HIF to the coactivator's p300/CREB-binding protein [62]. The activity of prolyl hydroxylase domains (PHDs) and FIHs is suppressed under hypoxic conditions. The heterodimeric HIF-*α*:HIF-1*β* transcription factor complex then translocates to the hypoxia-responsive elements (HREs) of its target genes, resulting in their transcriptional upregulation [63].

### 5.1. Brain Hypoxia and HIF-1*α*

The brain is a great energy consumer; therefore, it is particularly susceptible to hypoxia. Consequently, severe and prolonged oxygen deprivation can contribute to brain damage by inducing cell death and neurodegeneration. However, physiological responses to hypoxia are activated and mediated by HIF-1*α* for the cell to adapt to the microenvironment [64]. Transcriptional complex HIF-1*α*/*β* plays a crucial role in cellular and systemic oxygen homeostasis. This complex translocates into the nucleus, becoming a transcriptional activator of over 100 genes [65]. HIF-1*α* induces the transcription of vascular endothelial growth factor (VEGF), erythropoietin (EPO), and corresponding receptors (i.e., VEGF-R and EPO-R), promoting erythropoiesis and angiogenesis, thus increasing oxygen availability.

Furthermore, HIF-1*α* may also help in the activation of genes involved in glucose transportation and metabolism. Similarly, HIF-1*α* plays a vital role in maintaining homeostasis when oxygen deprivation occurs [5, 66].

### 5.2. Hypoxia Modulates the Accumulation of A*β* Peptides

Many studies have highlighted amyloid precursor protein (APP) and cleavage product A*β* in AD. APP is a single transmembrane protein expressed at high levels in the brain and is rapidly metabolized in a highly complex fashion by a series of sequential proteases [67]. The intramembranous *γ*-secretase complex also processes other key regulatory molecules [67]. Evidence supporting APP processing regulation spans the differentiation stages of cortical neurons, and amyloidogenic APP processing, as reflected by A*β*1-40/42, is associated with mature neuronal phenotypes [68]. Furthermore, genetic, biochemical, and behavioral investigations have also proved that physiologic generation of the neurotoxic A*β* peptide from sequential APP proteolysis is the crucial step in the development of AD [67]. Though the reason for the accumulation of A*β* in the brains of elderly individuals remains unclear, understanding the APP processing may be crucial to the development of therapeutic targets to treat AD. Hypoxia drives the metabolism of APP, leading to the amyloidogenic pathway, with A*β* protein as the end product [69]. Mattson [70] reported that this pathway could be a defense mechanism by increasing soluble neuroprotective APP*α* production. However, hypoxia favors APP metabolism through the amyloidogenic pathway, causing an increase in A*β* levels and not APP*α* levels [71]. Chronic hypoxia is shown to decrease the expression of disintegrin and metalloproteinase domain-containing protein 10 (ADAM10), a presumed *α*-secretase. Proteolytic processing of the APP by the *β*- and *γ*-secretases releases the A*β* peptide, which deposits in senile plaques and contributes to the etiology of AD. It also decreases APP cleavage through the nonamyloidogenic pathway [72, 73]. Hypoxia, respectively, decreases and increases the mature and immature forms of ADAM10 and reduces *α*-secretase processing of APP, which may represent a posttranslation effect [74]. However, studies have shown the decreased expression of ADAM17, an affiliated sheddase that also processes APP and TNF-*α*, after three days of chronic hypoxia [75]. *In vitro* studies of chronic hypoxia using cell lines and animal stroke models have shown increased *β*-secretase (BACE1) expression, an enzyme that increases the amyloidogenic pathway [76]. The positive feedback loop increases BACE1 levels and HIF-1*α*-induced genes, increasing amyloid-*β* protein production. This effect may occur because of the stabilization of HIF-1*α*, which A*β* also upregulates. Moreover, the fact that HIF-1*α*-deficient mice reduce BACE1 expression shows that HIF-1*α* is an essential mediator in BACE1 induction in hypoxic conditions [77].

### 5.3. Hypoxia, A*β* Accumulation, and Ca^2+^ Homeostasis

Hypoxia can cause a significant ionic disturbance because the ion channels are the first detector of a low oxygen level. During hypoxia, the intracellular ATP/ADP level ratio decreases, decreasing Na^+^/K^+^-ATPase activity and the influx of Ca^2+^, leading to membrane depolarization and increased intracellular Ca^2+^ [78]. As an intracellular ion, calcium ion is involved in several physiological processes, including neural excitability, second messenger signaling, and neurotransmitter release. However, excessive intracellular Ca^2+^ can cause changes to mitochondrial metabolism, activation of endonucleases, generation of ROS, and subsequent neurotoxicity [79]. Na^2+^-Ca^2+^ exchanger efflux pumps and buffering of the mitochondria and endoplasmic reticulum (ER) help maintain the homeostasis of intracellular Ca^2+^ in a healthy neuron [80]. However, excessive Ca^2+^ in neurons could result in the accumulation of amyloid-*β* protein. A possible mechanism for this accumulation is calcium-conducting pores formed by A*β* in the plasma membrane to regulate calcium ions in the neuron [81].

## 6. Kynurenine and Alzheimer's Disease: The Role of Indoleamine 2,3-Dioxygenase (IDO)

**Figure 3:**
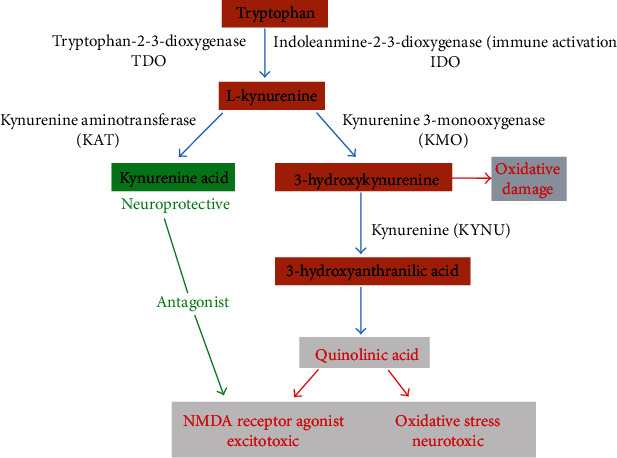
The kynurenine pathway. Breakdown of the kynurenine pathway in the brain as related to neurodegeneration. The four primary metabolites in the kynurenine pathway that readily cross the BBB are tryptophan, L-kynurenine, 3-hydroxykynurenine, and anthranilic acid. The metabolism of kynurenine metabolites in the brain occurs in two separate cells, microglial cells and astrocytes. In microglial cells, kynurenine is converted to 3-hydroxykynurenine, which causes oxidative damage and serves as the entry point of QA. QA is excitotoxic and neurotoxic. It acts as an agonist to the N-methyl-D-aspartate receptor and causes oxidative stress in the central nervous system. In astrocytes, L-kynurenine is converted to KA, an antagonist to the N-methyl-D-aspartate and alpha-7 nicotinic acetylcholine receptor. It also acts as a neuroprotective agent by blocking QA-induced neurodegeneration.

The kynurenine pathway involves the breakdown of tryptophan to nicotinamide adenine dinucleotide (NAD^+^) and other active metabolites. Tryptophan 2,3-dioxygenase (TDO) and indoleamine 2,3-dioxygenase (IDO) are the main enzymes involved in this pathway; they catalyze the rate-limiting step, which is the conversion of tryptophan to N-formylkynurenine (Figure 3) [82]. The cytosolic enzyme IDO is an endocellular, monomeric hemoprotein with a molecular mass of 45 kDa. Although first discovered in rabbit intestines, IDO expression occurs in the brain, kidney, lungs, spleen, and liver [83]. There are two isoforms of IDO: IDO1 and IDO2, which are widely expressed in various tissues. Although the gene coding for these isoforms is adjacent, they possess different biochemical properties and functions [83]. For instance, IDO2 expressed in the reproductive tract, kidney, colon, and liver has lower substrate specificity and expression levels than IDO1 [84]. The superior specificity of IDO1 marks it as a potential therapeutic target. The phosphorylation of IDO1 at tyrosine residues Y115 and Y253 helps to modulate its activity by changing IDO1 protein conformation, thus rendering it inactive [85].

Quinolinic acid (QA) and 3-hydroxykynurenine (3-HK) are also metabolites generated by the kynurenine pathway. These neurotoxic metabolites cause excitotoxicity and oxidative stress, respectively (Figure 3) [86, 87]. Furthermore, 3-HK aggravates neurodegeneration and contributes to AD development, while QA produced in the brain acts as an agonist and can induce oxidative stress [87, 88]. QA is also shown to be involved in tau protein phosphorylation by increasing synaptic and neuronal dysfunction [89]. The neuroprotective activity of kynurenic acid (KA) results from its antagonist effect on NMDA receptors [90]; hence, KA decreases QA-induced excitotoxicity. KA is an antagonist of the alpha-7 (*α*7) nicotinic receptors, reducing A*β*42 endocytosis, although the amount of KA produced is significantly lower than that of QA and 3-HK [91]. Interferon-gamma (INF-*γ*) and A*β*42 help stimulate the expression of IDO enzymes [92]. The overexpression of IDO enzymes can initiate different mechanisms in AD development, which may cause loss of neuronal activity and behavioral failure. More so, high levels of IDO are observed in the hippocampus of AD patients [7].

Besides, in experiments using a standard AD model, triple-transgenic AD (3xTg-AD) mice show high levels of INF-*γ* and IDO in their cerebrum [82]. The impairment caused by these high levels of INF-*γ* and IDO includes oxidative stress, increased levels of tau phosphorylation, impairment in the immune system, and increased A*β*42 levels. However, the role of TDO in the development of AD remains unclear. The TDO level is abundant in the liver despite being measured in the frontal cortex of patients with schizophrenia and at different levels in the mouse brain during their developmental phase [92, 93]. Wu et al. [94] showed that the hippocampi of patients with AD and 3xTg-AD mice presented with significantly elevated TDO levels in the cerebellum but not in the cerebrum. Thus, the kynurenine pathway might be a good target for AD treatment, as suggested by the increased levels of 3-HK and QA in the hippocampus and serum of patients with AD, respectively. A comparable elevation in QA was also noticed in 3xTg-AD mice [82, 95]. Evidence suggests that reducing the activity of the kynurenine pathway can mitigate some of the symptoms seen in experimental animal models of AD [96].

### 6.1. HIF-1*α* and the Kynurenine Pathway as Therapeutic Targets in Combating Neurodegenerative Disorders

The catabolism of tryptophan in the kynurenine pathway involves several enzymes that lead to the production of bioactive metabolites, including kynurenine. Kynurenine is involved in the modulation of the central nervous system and the immune system [97]. Due to this modulatory effect, kynurenine and some other metabolites from the kynurenine pathway are extensively studied for their usefulness in psychiatric, cancer, neuroinflammatory, and neurodegenerative diseases [98]. The kynurenine pathway is a shift to control inflammatory responses when there is a high level of inflammatory cytokines to produce KA, QA, anthranilic acid (AA), 3-hydroxyanthranilic acid (HAA), and 3-hydroxykynurenine (3-HK) [99, 100]. Among these metabolites, QA, 3-HK, and HAA are the neurotoxic metabolites generated. The level of 3-HK is excessively high in degenerative neural diseases like AD and Huntington's disease [97]. 3-Hydroxykynurenine is an oxidative stress generator, and its catabolism to HAA is an antitumor immunity highly beneficial for cancer treatment.

QA exhibits not only neurotoxicity but also neuroexcitatory effect [101]. Excess QA is produced during inflammation, and instead of being converted to NAD^+^ to protect the neurons, it gets saturated, resulting in lipid peroxidation and eventually ND. Moreover, AA shows immunomodulatory and anti-inflammatory activities [102], and together with HAA, 3-HK, and 3-HAA, they are all potent apoptotic agents [103]. A decrease in the plasma 3-HAA/AA ratio is seen in different NDs, which might be due to an increase in AA or a reduction in HAA [104]. According to Badawy [98], this ratio change might be an anti-inflammatory response or the presence and progression of inflammatory diseases. Although HIF-1*α* inhibits IDO, a key enzyme involved in the kynurenine pathway [105, 106], HIF-1*α* has implications in neuronal inflammatory disease pathology. It is responsible for the body's adaptation during hypoxia, and an altered HIF-1 *α* expression is reported in NDs [62, 107]. The accumulation and increase in HIF-1*α* attenuate the apoptosis caused via rotenone in PD by rescuing injured neurons [108].

**Figure 4:**
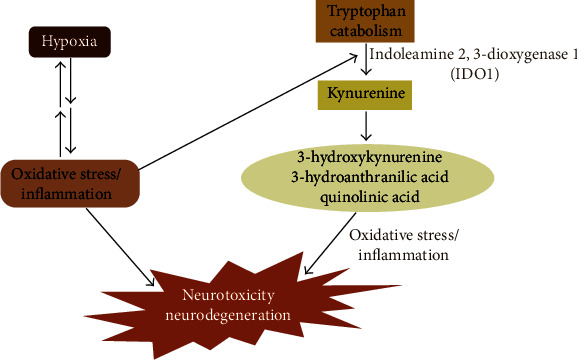
Diagram showing the effect of hypoxia and the kynurenine pathway on neurodegeneration.

Furthermore, energy or oxygen supply imbalance may activate various signaling mechanisms, including glutamatergic synapse formation, MAPK/PI3K-Akt signaling, and phosphatidylserine translocation, which play vital roles in oxidative stress and NDs. Indeed, HIF-1*α* plays a twofold role through gene activation, in the sense that this factor has to “choose” whether to protect or to kill the affected cells [109]. The regulation of HIF-1*α*, the exploration and internal control of the kynurenine pathway via the blockage or the expression of some critical metabolites, is a beneficial and potential medical target in neuroinflammatory and ND treatment. Taken together, the activation or inhibition of hypoxic intermediates with or without the kynurenine pathway metabolites could serve as novel therapeutic strategies for neurodegenerative disorders (Figure 4).

## 7. Conclusion

Hypoxia promotes the formation and accumulation of A*β*, which dysregulates calcium homeostasis in the neurons and astrocytes of the brain leading to neuronal loss or death and microglial activation. There is some evidence suggesting that APP cleavage alters the relationship between AD and hypoxia. This cleavage of APP leads to A*β* accumulation, an initial trigger of AD. Therefore, compounds with inhibitory potential against hypoxia and, in particular, HIF-1*α* may hold prospects in the development of neurodegeneration therapy. In addition, the rate-limiting enzyme in the kynurenine pathway, indoleamine 2,3-dioxygenase (IDO), produces two neurotoxic metabolites, 3-HK and QA, as end products of this pathway. When released locally in the brain, these metabolites can cause excitotoxic death to neurons and oligodendrocytes through their agonist effect on N-methyl-D-aspartic acid (NMDA) receptors. Therefore, compounds that restrict the kynurenine pathway activation may hold therapeutic prospects in neurodegenerative diseases such as AD. The typical therapies for AD, donepezil and galantamine, can only suppress or reduce AD symptoms but not as a cure to AD. Therefore, further *in vitro* and *in vivo* experiments are warranted to fully understand hypoxia and IDO enzyme roles on the kynurenine pathway to design novel therapeutic agents against ND, like AD.

## Data Availability

All data used have been reported in the article.
